# The impact of exposure to physical and sexual violence on opioid consequences among trauma-exposed individuals recruited from the community who use opioids

**DOI:** 10.1186/s12954-023-00901-y

**Published:** 2023-11-11

**Authors:** Prachi H. Bhuptani, Lindsay M. Orchowski, Shannon R. Forkus, Noam G. Newberger, Nicole H. Weiss

**Affiliations:** 1 Department of Adult Psychiatry, Rhode Island Hospital, Providence, RI USA; 2 Department of Psychiatry and Human Behavior, Brown University Medical School, Providence, RI USA; 3 Department of Psychology, University of Rhode Island, 142 Flagg Rd., Kingston, RI USA; 4 Medical University of South Carolina, Charleston, SC USA

**Keywords:** Interpersonal trauma, Sexual violence, Physical violence, Opioid

## Abstract

Interpersonal violence and opioid use disorder are significant and intersecting public health concerns in the USA. The current study evaluated the consequences associated with opioid use (e.g., physical, social, interpersonal, intrapersonal, and impulse control) as a function of a history of exposure to interpersonal trauma, specifically physical and sexual violence. Participants were 84 trauma-exposed individuals recruited from the community who use opioids (*M* age = 43.5 50% men; 55% white). Whereas no significant differences emerged in the consequences of opioid use based on a history of physical violence, individuals with a history of sexual violence demonstrated higher levels of impulsive consequences of opioid use compared to individuals without a history of sexual violence. These data highlight the importance of considering the role of exposure to sexual violence in the context of opioid use disorder treatment.

Opioid use disorder is a growing public health concern in the USA with high prevalence, morbidity, and mortality [1–3]. The Centers for Disease Control (CDC) estimated there have been more than 500,000 opioid overdose deaths in the USA over the past two decades [4]. Nationally, fatal opioid overdoses reached an all-time high in the USA in 2022, with more than 80,000 opioid-related overdose death reported [5]. Whereas opioid use is emerging as an epidemic, interpersonal trauma, which includes sexual and physical violence, has been documented as long as people have been recording history [6]. Sexual violence encompasses forcing or attempting to force a person to engage in sexual activity or touching and physical violence involves hurting, attempting to hurt, or threatening to hurt another person by use of physical force or weapons [7]. In the USA, both physical and sexual violence is highly prevalent. For example, a review of 249 articles revealed that in their lifetimes, 23.1% of women and 19.3% of men experienced physical violence [8]. The 2015 National Intimate Partner and Sexual Violence Survey (NISVS) revealed that 19.3% of women (out of 5,758) and 1.7% of men (out of 4,323) have been raped and 43.9% of women and 23.4% of men experienced other forms of sexual violence such as unwanted sexual contact [7].

These two public health concerns, namely opioid use and interpersonal trauma, are interrelated and bidirectional in nature. Prior research has highlighted the role of interpersonal trauma in contributing to problematic opioid use outcomes, including opioid use disorder and opioid overdose [9–11]. This can be explained by the opioid susceptibility model or self-medication model which posits that individuals with a history of interpersonal trauma may use opioids to cope with trauma-related psychological distress [e.g., posttraumatic stress disorder (PTSD)], physical pain and injuries, and psychosomatic symptoms (e.g., headaches, backpains), which increases their risk for developing opioid use disorder [12–17]. Opioid use also increases the risk of exposure to interpersonal trauma [18–23]. Contextual factors such as the decreased ability to assess risk when impaired by drugs, dependence on sexual partners for drug supply, coercion from an abusive partner to use drugs, and being forced to have sex in exchange for drugs or money, have all shown an increased risk for exposure to interpersonal trauma [20, 24–27]. Notably, the co-occurrence of opioid use and interpersonal trauma is marked by worsened clinical consequences associated with opioid use (e.g., exacerbations of psychological distress, increased opioid use), increased social consequences (e.g., legal, financial, and/or family problems), and poorer opioid use disorder treatment outcomes (e.g., higher rates of treatment drop out, more missed treatment appointments) [28–35].

Whereas extant research provides robust evidence for the relationship between opioid use and exposure to interpersonal trauma, there is an important gap. Namely, prior studies tended to limit their investigation to a single type of interpersonal trauma [e.g., only sexual violence [12, 20, 35, 36]]. When studies have examined multiple types of interpersonal trauma, they combined different types of interpersonal violence (such as sexual violence and physical violence) in a single composite variable [37–39]. This limits insight into whether different types of interpersonal trauma are differently related to consequences associated with opioid use. Nascent research has begun investigating the role of different types of trauma in the development and maintenance of opioid use disorder. For example, one study examined the impact of different types of interpersonal violence (i.e., intimate partner violence, sexual assault, and adverse childhood experiences) on problematic opioid use and found that only intimate partner violence and adverse childhood experiences were related to problematic opioid use [40]. Whereas this study examined different trauma types, they did not distinguish between physical and sexual violence. Two studies examined the pathways from childhood abuse to lifetime problematic opioid use among women and found that only sexual abuse—but not physical abuse, emotional abuse, or neglect—was associated with problematic opioid use [41, 42]. However, these are two studies, and thus there is a need for further investigation into the potentially differential impact of distinct types of interpersonal trauma.

## Present study

The current study investigated consequences associated with opioid use (i.e., physical, social, interpersonal, intrapersonal, and impulse control) based on participant’s history of exposure to interpersonal trauma, specifically physical and sexual violence. Consistent with the Diagnostic and Statistical Manual of Mental Disorders, Fifth Edition (DSM-5) [43] exposure to interpersonal trauma was defined as direct experiences, witnessing (in person) the event, learning the event happened to a close family member or friend, or experiencing repeated or extreme exposure to aversive details of the event(s) (e.g., first responders). Given that opioid use and interpersonal trauma co-occur at high rates [10, 12] leads to worse outcomes [28, 29, 32, 35], and different types of trauma may be more relevant [41, 42] it was hypothesized as follows:

### Hypothesis 1

Individuals with a history of exposure to physical violence would report significantly more consequences of opioid use, compared to those without a history of exposure to physical violence.

### Hypothesis 2

Individuals with a history of exposure to sexual violence would report significantly more consequences of opioid use, compared to those without a history of exposure to sexual violence.

## Methods

### Participants

Participants were recruited from the Providence metropolitan area, an urban region anchored by the city of Providence, Rhode Island with a population of greater than 1.6 million. Recruitment materials were posted in community establishments throughout Providence County, Rhode Island including grocery stores, laundromats, and shops; selected state offices such as the Office of Housing and Community Development; and waiting rooms, bathrooms, and examination rooms of urban-area primary care clinics; as well as in website postings (e.g., Craigslist). Further, research assistants recruited at/alongside local harm reduction agencies (e.g., street outreach, warming centers) that serve individuals who use opioids (e.g., needle exchange). Eligibility was determined through self-report during a phone or in-person screen. Participants were individuals who had experienced trauma in their lifetime and used illegal opioids (e.g., heroin) or misused prescription opioids (i.e., used prescription opioids without a prescription or in a manner not prescribed such as taking a higher dose than prescribed or for a longer period than prescribed) during the past 30 days. Specifically, Item 1 of the Primary Care PTSD Screen for DSM-5 [44] was used to assess past 30-day trauma exposure. Specifically, participants were asked: “Sometimes things happen to people that are unusually or especially frightening, horrible, or traumatic. For example, a serious accident or fire, a physical or sexual assault or abuse, an earthquake or flood, a war, seeing someone be killed or seriously injured, having a loved one die through homicide or suicide. Have you ever experienced this kind of event?” Further, three items were administered to assess past 30-day opioid use. Specifically, participants were asked: “In the last month, did you use (1) opioids, (2) prescription pain relievers such as oxycodone, hydrocodone, codeine, or morphine that were not prescribed to you or use prescription drugs not as prescribed in order to feel the effects? (e.g., you took more than prescribed or took them for a longer time than prescribed); or (3) synthetic opioids like fentanyl that were not prescribed to you or use prescription drugs not as prescribed in order to feel the effects? (e.g., you took more than prescribed or took them for a longer time than prescribed).” If they answered yes to opioid items 1, 2, or 3, they were eligible for the study. One-hundred and sixty individuals called to learn more about the study, two of whom were not interested with proceeding with screening questions after learning what the study entailed. Of the people who were screened (*n* = 158), 41 were not eligible. Of the eligible participants (*n* = 117), two were not interested in being scheduled after they were screened. Thus, 115 participants were eligible and scheduled. Of the participants who were scheduled, 31 participants dropped out prior to consent (baseline session), leaving 84 participants in the final sample.

Additional inclusion criteria were: (1) age 18 or older, (2) fluent in the English language, and (3) owning a smartphone. Exclusion criteria were (a) current mania/psychosis (assessed in the baseline session with the Structured Clinical Interview for DSM-V [SCID-5]; First and Williams, 2016) and (b) current impairment in cognitive functioning (assessed in the baseline session using the mini-mental status examination and requiring a score > 24; Folstein et al., 1975). The sample reported here included 84 individuals who participated in a baseline session; demographic characteristics are summarized in Table 1.

**Table 1:** Demographic summary

	*M* (SD)	*N* (%)
Age	43.45 (11.06)	
*Gender*		
Men		42 (50.0%)
Women		35 (41.7%)
Transgender		3 (3.6%)
Gender queer/non-binary		1 (1.2%)
Prefer not to respond		3 (3.6%)
*Race*		
White		46 (54.8%)
Black/African American		17 (20.0%)
multiracial		8 (9.5%)
American Indian/Alaskan native		3 (3.6%)
Native Hawaiian/other Pacific Islander		1 (1.2%)
Prefer not to respond		9 (10.7%)
*Ethnicity*		
Not Hispanic or latinx		61 (72.6%)
Hispanic or latinx		9 (10.7%)
Prefer not to respond		14 (16.7%)
*Sexual orientation*		
Heterosexual		63 (75.0%)
Bisexual		10 (11.9%)
Lesbian/Gay		3 (3.6%)
Pansexual		1 (1.2%)
Unsure		1 (1.2%)
Prefer not to respond		6 (7.1%)
Income	$948.63 ($852.09)	
*Employment status*		
Unemployed		46 (54.8%)
Part time (Less than 35 h per week or sporadic employment)		13 (15.5%)
Not in labor force (e.g., student, homemaker)		11 (13.1%)
Full time (More than 35 h per week)		5 (6.0%)
Prefer not to respond		9 (10.7%)
*Relationship status*		
Seriously dating (I do not date other people)		25 (29.8%)
Not dating		23 (27.4%)
Casually dating (I date other people as well)		11 (13.1%)
Separated		7 (8.3%)
Married		7 (8.3%)
Divorced		4 (4.8%)
Widowed		2 (2.4%)
Prefer not to respond		5 (6.0%)

### Procedures

All procedures were reviewed and approved by the [redacted] Institutional Review Board. The larger study entailed (a) a baseline session, (b) 30 days of ecological momentary assessment (five surveys per day) on a smartphone app, and (c) a follow-up session. The current study used data from the baseline session. Baseline sessions were conducted by a clinical psychology doctoral student in a private office to protect participants’ safety and confidentiality. After providing informed consent, participants were interviewed using a structured diagnostic assessment and then answered self-report measures on a computer. Participants were compensated with $25 for completing the baseline session. Participants were provided with a list of community resources. Assistance with referrals was provided upon participant request. The principal investigator (author [redacted]), a licensed psychologist in the state of Rhode Island, was available on-call if participants required additional trauma- and/or substance-related support.

### Measures

#### Interpersonal trauma

The 17-item Life Events Checklist for DSM-5 [LEC-5; [45]] was used to assess a history of exposure to physical violence or sexual violence. Participants rated each time with six response options: happened to me, witnessed it, learned about it, part of my job, not sure, or doesn’t apply. Specifically, items “Assault with a weapon (for example, being shot, stabbed, threatened with a knife, gun, bomb)” and “Physical assault (for example, being attacked, hit, slapped, kicked, beaten up)” were used to measure the experience of exposure to physical violence, whereas “Sexual assault (rape, attempted rape, made to perform any type of sexual act through force or threat of harm)” and “Other unwanted or uncomfortable sexual experience” were used to measure the experience of exposure to sexual violence. For the current study, exposure to physical or sexual violence was indicated when participants selected either “happened to me,” “witnessed it,” “learned about it,” or “part of my job,” as consistent with Criterion A for posttraumatic stress disorder in the Diagnostic Statistical Manual of Mental Disorder, Edition 5 [DSM-5 [46]].

#### Consequences associated with opioid use

The 17-item Short Inventory of Problems Scale-Revised [SIPR; [47]] was adapted to assess consequences associated with opioid use. The scale has five subscales: physical consequences (“Because of my opioid use, I have lost weight or not eaten properly”), social consequences ("I have failed to do what is expected of me because of my opioid use”), intrapersonal consequences ("I have felt guilt or ashamed because of my opioid use”), impulse control (“I have taken foolish risks when I have been using opioids”), and interpersonal consequences (“My family has been hurt by my opioid use”). Participants responded on a 4-point Likert scale, ranging from 0 *(Never)* to 3 (*Daily or Almost Daily*). Items were summed and higher scores indicated more frequently experienced problems associated with opioid use. The internal consistency of all five subscales ranged from good to excellent. McDonald’s Omega was as follows: 0.84 (physical consequences), 0.93 (social consequences), 0.89 (intrapersonal consequences), 0.89 (interpersonal consequences), and 0.93 (impulsive consequences).

#### Data analysis

Descriptive data for the primary study variables were calculated. In order to examine whether consequences of opioid use varied across history of exposure to physical violence, one-way ANOVA was conducted where a history of exposure to physical violence (0 = no history of exposure to physical violence, 1 = history of exposure to physical violence) was entered as the independent variable, and consequences of opioid use (i.e., physical, social, intrapersonal, interpersonal, and impulsive) were entered as dependent variables. Similarly, to examine whether consequences of opioid use varied across a history of exposure to sexual violence, one-way ANOVA was conducted where a history of exposure to sexual violence (0 = no history of exposure to sexual violence, 1 = history of exposure to sexual violence) was entered as the independent variable and consequences of opioid use (i.e., physical, social, intrapersonal, interpersonal, and impulsive) were entered as dependent variables.

## Results

### Preliminary analyses

When examining past-month opioid use in the current sample, 59.5% (*n* = 50) reported using heroin, 70.2% (*n* = 59) reported using prescription opioids without a prescription or in a manner not prescribed such as taking a higher dose than prescribed or for a longer period than prescribed, and 82.1% (*n* = 69) reported using synthetic opioids (e.g., Fentanyl). All participants in the current study reported a history of trauma. When examining exposure to interpersonal violence in the current sample, over half of the sample reported a history of exposure to physical (*n* = 56; 66.7%) *or* sexual (*n* = 47; 56.0%) violence. Over half of the sample, (*n* = 45, 53.57%) endorsed exposure to both physical *and* sexual violence. Further details regarding the prevalence of history of exposure to physical and sexual violence are summarized in Table 2.

**Table 2:** Prevalence of interpersonal trauma

	***N***	**%**
Physical assault (e.g., being attached, hit, slapped, kicked, beaten up)		
Criterion A trauma met^a^	52	61.9%
Happened to me	46	54.8%
Witnessed it	3	3.6%
Learned about it	0	0
Part of my job	3	3.6%
Not sure	2	2.4%
Does not apply	16	19.0%
Prefer not to respond	14	16.7
Assault with a Weapon (e.g., being shot, stabbed, threatened with a knife, gun bomb)		
Criterion A trauma met^a^	45	53.6%
Happened to me	36	42.9%
Witnessed it	4	4.8%
Learned about it	2	2.4%
Part of my job	3	3.6%
Not sure	3	3.6%
Does not apply	24	28.6%
Prefer not to respond	12	14.3%
Sexual Assault (e.g., rape, attempted rape, sexual act through force or threat of harm)		
Criterion A trauma met^a^	38	45.3%
Happened to me	33	39.3%
Witnessed it	3	3.6%
Learned about it	1	1.2%
Part of my job	1	1.2%
Not sure	4	4.8%
Does not apply	30	35.7%
Prefer not to respond	12	14.3%
Other unwanted or uncomfortable sexual experience		
Criterion A trauma met^a^	42	50.0%
Happened to me	33	39.3%
Witnessed it	5	6.0%
Learned about it	1	1.2%
Part of my job	3	3.6%
Not sure	2	2.4%
Does not apply	29	34.5%
Prefer not to respond	11	13.1%

^a^Participants endorsed happened to me, witnessed it, learned about it, or part of my job

### Impact of exposure to physical and sexual violence

See Table 3 for one-way ANOVA tests examining consequences of opioid use as a function of history of exposure to physical and sexual violence. No significant differences emerged in the physical, social, intrapersonal, interpersonal, or impulsive consequences of opioid use based on a history of exposure to physical violence. Conversely, individuals with a history of exposure to sexual violence demonstrated significantly higher levels of impulsive (*M* = 5.34, *SD* = 3.44)—but not physical, social, intrapersonal, or interpersonal—consequences compared to individuals without a history of exposure to sexual violence (*M* = 3.53, SD = 3.06).

**Table 3:** Differences in consequences of opioid use by history of exposure to physical and sexual violence

	*M* (SD)	*F*	*p*	Eta-Squared	
Physical Violence					
	No History of Physical Violence (*n* = 28)	History of Physical Violence (*n* = 56)			
SIPR Physical	3.60 (3.20)	4.53 (3.17)	1.27	.26	.01
SIPR Intrapersonal	4.00 (3.21)	5.56 (3.19)	3.09	.08	.04
SIPR Social	6.05 (5.57)	7.77 (5.22)	1.54	.22	.02
SIPR Interpersonal	3.80 (3.41)	3.41 (3.60)	1.13	.29	.02
SIPR Impulsive	3.63 (3.32)	5.02 (3.81)	2.40	.12	.03
Sexual violence					
	No History of Sexual Violence (*n* = 37)	History of Sexual Violence (*n* = 47)			
SIPR Physical	3.52 (2.75)	4.77 (3.37)	2.82	.10	.04
SIPR Intrapersonal	4.17 (3.12)	5.64 (3.22)	3.81	.05	.05
SIPR Social	6.28 (4.95)	7.95 (5.51)	1.80	.18	.02
SIPR Interpersonal	3.72 (3.09)	4.98 (3.49)	2.48	.12	.03
**SIPR Impulsive**	**3.53 (3.06)**	**5.34 (3.44)**	5.23	**.02**	**.07**

Bolded consequence is significant at *p* < .05

## Discussion

The current study investigated the unique impact of exposure to physical and sexual violence on consequences associated with opioid use among trauma-exposed individuals recruited from the community who use opioids. The link between exposure to interpersonal trauma and increased consequences associated with opioid use has been well established in prior literature [28, 29, 32, 35]. To our knowledge, this is the first study to examine whether the consequences of opioid use differed based on participant’s history of exposure to interpersonal trauma, specifically physical and sexual violence.

Contrary to our hypothesis, individuals with a history of exposure to physical violence reported similar levels of consequences associated with opioid use, compared to individuals without a history of exposure to physical violence. Our second hypothesis, specifically, individuals with a history of exposure to sexual violence would report higher levels of consequences associated with opioid use, compared to individuals without a history of exposure to sexual violence, was partially supported. Specifically, results suggested that individuals with a history of exposure to sexual violence demonstrated significantly high levels of impulsive consequences—but not physical, social, intrapersonal, or interpersonal consequences—of opioid use compared to individuals without a history of exposure to sexual violence. Results are in line with prior studies that indicate sexual violence, compared to other types of violence, is particularly detrimental, even when compared to physical violence [48, 49]. It warrants mention that the current sample consisted of all trauma-exposed individuals. This history of exposure to trauma may explain why most of the consequences of opioid use, except for impulsive consequences, did not differ among individuals who did and did not experience exposure to physical or sexual violence. Future research is needed to compare individuals with physical and sexual violence to those without any history of trauma on their consequences of opioid use.

Notably, results highlight the particularly adverse impact of exposure to sexual violence on impulsive consequences of opioid use. The link between a history of exposure to sexual violence and consequences associated with opioid use, especially impulsive consequences, may be understood through the lens of emotion dysregulation. Emotion dysregulation is a multifaceted construct that refers to difficulties understanding and modulating emotions [50]. Robust evidence shows that individuals with a history of exposure to sexual violence demonstrate higher levels of emotion dysregulation compared to those without a history of exposure to sexual violence [51–53]. Emotion dysregulation, in turn, is also associated with impulsive behaviors [54–56], including among individuals with a history of exposure to sexual violence [52, 57, 58]. Further, deficits in emotion regulation among individuals with a history of exposure to sexual violence have also been linked to greater problematic substance use [for a review, see [59]], including opioid use [12, 60], which is consistent with the self-medication/opioid susceptibility hypothesis [17, 61]. Collectively, findings suggest the utility of targeting emotion dysregulation to address impulsivity consequences of opioid use among individuals with a history of exposure to sexual violence [for a review see [62]].

Another possible reason that may explain the findings of current study is the link between PTSD and impulsivity. Individuals exposed to sexual violence are at an exponentially greater risk for developing PTSD, compared to other types of trauma (e.g., physical assault) [63–65]. Further, robust evidence has linked PTSD symptoms to heightened impulsivity [66, 67], and heightened impulsivity with increased substance use [68, 69]. Although not assessed here, individuals exposed to sexual violence in the current study may be at an increased risk for using opioids due to increased PTSD symptoms and the impact of PTSD on impulsivity. Subsequently, opioid use may then exacerbate impulse control consequences. Indeed, one study compared levels of impulsivity among individuals with concurrent OUD and PTSD, OUD without PTSD, PTSD without OUD, and individuals without OUD or PTSD [70]. The authors found that the concurrent OUD and PTSD, OUD without PTSD, and PTSD without OUD groups reported higher levels of impulsivity compared to individuals without OUD or PTSD. Additionally, the authors also found that the concurrent OUD and PTSD group also reported greater levels of impulsivity compared to the OUD without PTSD group and individuals without OUD or PTSD but not the PTSD without OUD group. Thus, the authors’ concluded that impulsivity mechanism links OUD and PTSD which may explain why individuals exposed to sexual violence in the current study, who are at a greater risk for experiencing PTSD, may also show greater levels of impulse control consequences related to opioid use.

Study results have several implications for clinical practice and research. Given the detrimental impact of comorbid sexual violence and problematic opioid use, clinicians should incorporate regular screening for exposure to sexual violence in treatment for opioid use disorder. Given that many individuals exposed to sexual violence may delay disclosure of violence to treatment providers [71], and problematic opioid use is associated with increased risk for exposure to sexual violence [20], it is important for clinicians to routinely screen for exposure to sexual violence. Further efforts need to be targeted toward the development and evaluation of interventions aimed at concomitantly reducing exposure to sexual violence and problematic opioid use to effect a change in harmful effects of both exposure to sexual violence and opioid use. Such efforts can be built on prior work that has already been done on alcohol and its relationship to exposure to sexual violence while heeding to unique differences that may exist between alcohol-involved exposure to sexual violence and opioid-involved exposure to sexual violence [72]. Given the bidirectional nature of exposure to sexual violence and problematic opioid use, interventions targeting opioid use among individuals exposed to sexual violence also need to be developed and evaluated [36]. Finally, the results highlight the need to incorporate a trauma-informed approach in care and treatment for opioid use disorder such as fostering collaboration, maximizing client’s choice and control, emphasizing client’s strengths, and creating a safe atmosphere [73].

### Limitations and future directions

The results of the current study should be interpreted in the context of several limitations, which also pave the way for future directions. First, our relatively small sample size limits investigation into gender differences, polyvictimization (i.e., experiencing more than one type of interpersonal violence), and revictimization (i.e., repeated occurrences of interpersonal violence). Preliminary research suggests gender differences in the relation between interpersonal violence and opioid use [40, 42], and both polyvictimization and revictimization are associated with increased substance use [74, 75]. Thus, future studies with larger sample size should determine the role of gender, polyvictimization, and revictimization in the relations between both exposure to physical and sexual violence and problematic opioid use. Second, given that cross-sectional findings preclude temporal interpretations, future longitudinal studies with multiple time points are needed to establish the likely cyclical relation between exposure to sexual violence and problematic opioid use. Finally, findings cannot be assumed to generalize to other populations characterized by opioid use, including individuals seeking outpatient or residential treatment for problematic opioid use. Thus, findings require replication across other populations that use opioids.

### Conclusion

The current study investigated differences in consequences associated with opioid use depending on history of exposure to interpersonal trauma, particularly physical and sexual violence, among trauma-exposed individuals recruited from the community who use opioids. Results suggest that individuals with a history of exposure to sexual violence in particular demonstrated a higher level of impulsive consequences associated with opioid use. Findings emphasize the need to concomitantly address both sexual violence and problematic opioid use.

## Data Availability

The datasets used and/or analyzed during the current study are available from the corresponding author upon reasonable request.

## Abbreviations

CDC: Centers for disease control
PTSD: Posttraumatic stress disorder
SCID-5: Structured clinical interview for DSM-V
DSM-5: Diagnostic statistical manual of mental disorder, edition 5
LEC-5: Life events checklist for DSM-5
SIPR: Short inventory of problems scale-revised
